# Myeloid and CD4 T Cells Comprise the Latent Reservoir in Antiretroviral Therapy-Suppressed SIVmac251-Infected Macaques

**DOI:** 10.1128/mBio.01659-19

**Published:** 2019-08-20

**Authors:** Celina M. Abreu, Rebecca T. Veenhuis, Claudia R. Avalos, Shelby Graham, Daymond R. Parrilla, Edna A. Ferreira, Suzanne E. Queen, Erin N. Shirk, Brandon T. Bullock, Ming Li, Kelly A. Metcalf Pate, Sarah E. Beck, Lisa M. Mangus, Joseph L. Mankowski, Feilim Mac Gabhann, Shelby L. O’Connor, Lucio Gama, Janice E. Clements

**Affiliations:** a Department of Molecular and Comparative Pathobiology, Johns Hopkins School of Medicine, Baltimore, Maryland, USA; b Department of Pathology, Johns Hopkins School of Medicine, Baltimore, Maryland, USA; c Department of Neurology, Johns Hopkins School of Medicine, Baltimore, Maryland, USA; d Department of Biomedical Engineering, Johns Hopkins University, Baltimore, Maryland, USA; e Institute for Computational Medicine, Johns Hopkins University, Baltimore, Maryland, USA; f Department of Pathology and Laboratory Medicine, University of Wisconsin—Madison, Madison, Wisconsin, USA; g Vaccine Research Center, National Institute of Allergy and Infectious Diseases, Bethesda, Maryland, USA; Emory University; National Institute of Allergy and Infectious Diseases

**Keywords:** HIV, latency, SIV, macrophages, monocytes

## Abstract

Human immunodeficiency virus (HIV) eradication or long-term suppression in the absence of antiretroviral therapy (ART) requires an understanding of all viral reservoirs that could contribute to viral rebound after ART interruption. CD4 T cells (CD4s) are recognized as the predominant reservoir in HIV type 1 (HIV-1)-infected individuals. However, macrophages are also infected by HIV-1 and simian immunodeficiency virus (SIV) during acute infection and may persist throughout ART, contributing to the size of the latent reservoir. We sought to determine whether tissue macrophages contribute to the SIVmac251 reservoir in suppressed macaques. Using cell-specific quantitative viral outgrowth assays (CD4-QVOA and MΦ-QVOA), we measured functional latent reservoirs in CD4s and macrophages in ART-suppressed SIVmac251-infected macaques. Spleen, lung, and brain in all suppressed animals contained latently infected macrophages, undetectable or low-level SIV RNA, and detectable SIV DNA. Silent viral genomes with potential for reactivation and viral spread were also identified in blood monocytes, although these cells might not be considered reservoirs due to their short life span. Additionally, virus produced in the MΦ-QVOA was capable of infecting healthy activated CD4s. Our results strongly suggest that functional latent reservoirs in CD4s and macrophages can contribute to viral rebound and reestablishment of productive infection after ART interruption. These findings should be considered in the design and implementation of future HIV cure strategies.

## Abstract (precis)

This study provides further evidence that the latent reservoir is comprised of both CD4^+^ T cells and myeloid cells. The data presented here suggest that CD4^+^ T cells and macrophages found throughout tissues in the body can contain replication-competent SIV and contribute to rebound of the virus after treatment interruption. Additionally, we have shown that monocytes in blood contain latent virus and, though not considered a reservoir themselves due to their short life span, could contribute to the size of the latent reservoir upon entering the tissue and differentiating into long-lived macrophages. These new insights into the size and location of the SIV reservoir using a model that is heavily studied in the HIV field could have great implications for HIV-infected individuals and should be taken into consideration with the development of future HIV cure strategies.

## INTRODUCTION

Identifying the viral reservoir is critical for designing and testing human immunodeficiency virus (HIV) strategies to eliminate or fully suppress the virus. Currently, the latent HIV type 1 (HIV-1) reservoir found in CD4 T cells is measured by assays that quantify the functional latent reservoir, the primary target of HIV eradication studies. It is well recognized that HIV infects monocytes and macrophages and that these myeloid cells contribute to disease progression. However, myeloid cells have not been systematically examined as a latent reservoir in antiretroviral therapy (ART)-suppressed individuals. Therefore, it is not clear whether myeloid cells constitute an additional viral reservoir that must be considered in eradication strategies. The HIV-1 CD4 T cell reservoir in blood has been shown to be long lived using a quantitative viral outgrowth assay (QVOA). This assay estimates the number of cells that harbor replication-competent viral genomes that could contribute to viral rebound after ART interruption. The QVOA has been used to demonstrate the stability as well as the decay rate of the CD4 T cell reservoir (1, 2). Here we demonstrate for the first time in ART-suppressed SIVmac251-infected rhesus macaques that CD4 T cells, monocytes, and tissue macrophages harbor latent, replication-competent viral genomes using CD4 T cell QVOA and macrophage QVOA (MΦ-QVOA) assays previously developed by our laboratory (3–5).

In the absence of ART, HIV-1 infection results in depletion of CD4 T cells and immunosuppression. Infection of monocytes and macrophages causes organ-specific diseases in the brain, lung, heart, and gut (6–10). Macrophages are the primary targets of productive HIV-1 infection in brain and lung, and viral RNA can be detected in these cells by *in situ* hybridization (ISH) in HIV-infected individuals (11, 12). Similarly, in simian immunodeficiency virus (SIV)-infected macaques, SIV RNA and DNA can be measured in macrophages in brain, lung, and spleen during infection (3, 13–18).

In the era of ART, the role of myeloid cells as viral reservoirs or as contributors to ongoing HIV-1 morbidity has mainly been evaluated using the brains of patients with HIV-associated neurocognitive diseases (HAND) (19–21). Postmortem studies of brains from HAND patients have demonstrated the presence of HIV-1 DNA (16, 22), and ongoing low-level inflammation in central nervous system (CNS) myeloid cells has been suggested to contribute to morbidity (22–24). The “Boston Patients,” who were given bone marrow transplants and remained HIV-1 negative for months after stopping ART, experienced HIV rebound in blood and CNS. HIV-1 was detected in cerebrospinal fluid (CSF) in both patients, and one patient experienced CNS symptoms before HIV-1 rebound in the blood (25, 26). These studies implicate myeloid cells as HIV-1 reservoirs in brain and suggest that myeloid cells in other sites may also harbor functional latent HIV-1 reservoirs.

The SIVmac251 rhesus macaque model of HIV-1 infection and pathogenesis has been one of the most frequently used models to study HIV-1 viral pathogenesis, vaccine research, drug development, and eradication (27–34). The model recapitulates HIV-1 infection and progression to AIDS in humans as well as infection of myeloid cells in the CNS (35–37). However, SIVmac251-infected macaques rarely develop classic SIV encephalitis unless CD8^+^ T cells are depleted (20, 38). In addition, when CD4 T cells are depleted in macaques prior to infection with SIVmac251, the infection results in high viral load, infection of myeloid cells in the brain, and the development of encephalitis (33). These studies provide evidence that SIVmac251 does infect myeloid cells in macaques and establish that these cells play an important role in SIV pathogenesis. Thus, the SIVmac251 rhesus macaque model provides an appropriate model to investigate the role of latency in myeloid cells during ART.

It is critical to employ rigorous latency assays currently used in HIV-1 studies to assess the functional latent viral reservoir in the SIVmac251 rhesus macaque model. An SIV resting CD4 T cell QVOA was previously developed in our laboratory (39). We demonstrated in a SIV-infected macaque model of AIDS and suppressive ART that the frequency of latently infected resting CD4 T cells in blood and lymph nodes was one infected cell per million CD4 T cells, which is the same frequency observed in ART-suppressed HIV-infected individuals (1, 2, 39). In addition, we have recently reported the development of a quantitative viral outgrowth assay for myeloid cells, the MΦ-QVOA. The MΦ-QVOA was used to quantitate the frequency of latently infected brain (3), spleen, lung and blood (5) macrophages in macaques suppressed on ART for 1 to 1.5 years. Latently infected brain macrophages were identified in 70% of ART-suppressed macaques, whereas latently infected spleen, lung, and blood macrophages were identified in 100% of ART-suppressed macaques assessed. These studies demonstrated that macrophages in SIV-infected ART-suppressed macaques represent a functional latent reservoir that harbors replication-competent virus, a potential barrier to viral eradication.

This study measures CD4 T cell and macrophage functional latent reservoirs for the first time in the SIVmac251 rhesus macaque model during ART suppression. SIV DNA was detected in all tissue samples in the ART-suppressed macaques, including blood monocytes, which also carried genomes that could be reactivated *ex vivo*. Additionally, all ART-suppressed SIVmac251-infected macaques harbored functional latent tissue macrophages. Finally, viruses produced in the MΦ-QVOAs were replication competent and capable of producing *de novo* infection of CD4 T cells. This is the first study to measure the functional latent reservoir in ART-suppressed SIVmac251 rhesus macaques and establishes the level of functional latency in both CD4 T cells and myeloid cells.

## RESULTS

### Characteristics of untreated and ART-suppressed SIVmac251-infected macaques.

**FIG 1:**
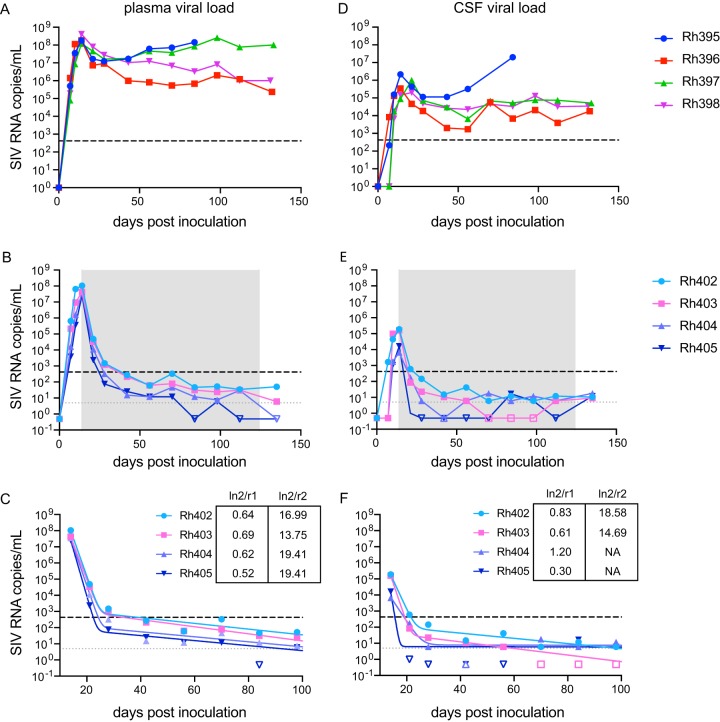
Viral load in plasma and CSF in untreated and ART-suppressed SIVmac251-infected macaques. Eight rhesus macaques were infected with SIVmac251; four were left untreated, and four were treated with ART starting at 14 dpi. Viral load was measured longitudinally in the plasma and CSF samples from the untreated (A and D) and ART-treated (B and E) groups. Decay from peak viremia in plasma (C) and CSF (F) for the four ART-treated animals was determined using a biphasic two-exponential decay model. Solid lines indicate the best-fit biphasic model for each animal. Graphs display two limits of detection (dashed lines), depending on the assay, as described in the text; filled symbols indicate measurements above the limit of quantitation for that measurement; open symbols indicate measurements below the limit of quantitation. Insets display half-lives for both phases of decay.

Our laboratory chose the SIVmac251 rhesus macaque model to examine viral latent reservoirs due to its acceptance as a model of HIV-1 infection. In order to characterize both the peripheral reservoir as well as the reservoir in the CNS, longitudinal viral load was measured in both plasma and CSF. The CSF viral load has been rarely measured in this SIV model without immune modulation (20, 40–43). However, a previously published study that did monitor viral load in the CSF without immune modulation found virus during acute infection (44), and understanding the level of virus in the CSF is essential for monitoring CNS infection. Eight rhesus macaques were inoculated intravenously with SIVmac251. SIV RNA was measured longitudinally in plasma and uniformly detected in all animals by day 7 postinoculation (p.i.) (Fig. 1A and B). The peak levels of plasma viremia occurred at day 14 p.i. in all animals (median, 1.09 × 10^8^ SIV RNA copies/ml; range, 2.58 × 10^7^ to 4.10 × 10^8^ copies/ml). SIV RNA was initially detected in CSF at day 7 p.i. for three animals and at day 10 p.i. for five animals (Fig. 1D and E). Similar to the results for plasma, all eight animals demonstrated peak levels of CSF viral load by day 14 p.i. (median, 1.09 × 10^5^ SIV RNA copies/ml; range, 6.66 × 10^3^ to 2.17 × 10^6^ copies/ml). The lack of blood cells in CSF samples, analyzed by Cytospin (45), indicated that SIV detected in the CNS viremia had originated in the CNS and was not a result of contamination from the plasma during collection.

Of the eight SIV-infected animals, four remained untreated until necropsy at 130 days p.i., and four were treated with ART starting at day 14 p.i. All untreated animals maintained stable levels of plasma viremia until necropsy, with one animal euthanized early due to non-SIV-related complications (Fig. 1A and Table 1). Similar to the results for plasma, the four SIV-infected untreated animals experienced only a slight decay in viral load in CSF from peak at day 14 p.i. to a set point viral load that was maintained above 1,000 copies/ml. SIV infection measured by SIV RNA in plasma in these macaques was comparable to other SIVmac251 rhesus studies (28, 46). However, there are limited data on CSF viral load in this model, without manipulation of the immune system, available for comparison. We found that three of the four untreated macaques maintained stable viral loads in the CSF, and that in one animal, SIV RNA levels continued to increase until necropsy (Fig. 1D).

**TABLE 1:** Detailed characterization of SIV-infected macaques used in the study before ART initiation and at necropsy (terminal time point)^*a*^

Group and animal	Pre-ART initiation					Necropsy				
	dpi	Cell count (cells/μl)		Viral load (SIV copies/ml)		dpi	Cell count (cells/μl)		Viral load (SIV copies/ml)	
		CD4^+^ T cells	Monocytes	Plasma	CSF		CD4^+^ T cells	Monocytes	Plasma	CSF
Untreated										
Rh395	14	453	970	1.8 × 10^8^	2.2 × 10^6^	98	253	1,050	1.6 × 10^8^	1.4 × 10^7^
Rh396	14	561	960	1.8 × 10^8^	3.4 × 10^5^	132	318	500	2.4 × 10^5^	1.8 × 10^4^
Rh397	14	396	370	1.2 × 10^8^	8.9 × 10^4^	133	1,240	850	1.0 × 10^8^	5.2 × 10^4^
Rh398	14	563	460	4.1 × 10^8^	1.5 × 10^5^	131	395	530	1.0 × 10^6^	3.5 × 10^4^

ART-suppressed										
Rh402	14	754	1,140	1.1 × 10^8^	1.9 × 10^5^	132	1,187	460	51	10
Rh403	14	773	490	4.3 × 10^7^	1.6 × 10^5^	134	1,149	530	6	9
Rh404	14	234	330	3.5 × 10^7^	6.7 × 10^4^	133	375	420	<LOD	18
Rh405	14	535	320	2.6 × 10^7^	1.6 × 10^4^	135	1,087	450	<LOD	12

a Abbreviations: dpi, days postinoculation; LOD, limit of detection (5 copies/ml).

The four ART-treated animals all displayed rapid decay of plasma viremia to below the limit of quantitation (LOQ) of our quantitative reverse transcription-PCR (qRT-PCR) assay (420 RNA copies/ml) by day 42 p.i., 28 days after ART initiation (Fig. 1B). To increase the sensitivity of SIV RNA detection, all samples that were at or below the LOQ of the qRT-PCR assay were also measured by digital droplet PCR (ddPCR) (LOQ, 5 copies/ml). From day 42 p.i. to necropsy at 130 p.i., six viral load measurements were taken for each suppressed animal (Fig. 1B). All viral loads were below 50 copies/ml (LOD of standard clinical assays) as measured by ddPCR by day 84 p.i., 70 days after ART initiation. As in plasma in the four ART-suppressed animals, SIV RNA showed a rapid decay in CSF to below the qRT-PCR LOQ by day 28 p.i. As with plasma, SIV RNA in all CSF samples at or below the qRT-PCR LOQ were also tested by ddPCR. From day 28 p.i. to necropsy at day 130 p.i., there were seven viral load measurements, all of which were below 50 copies/ml as measured by ddPCR (Fig. 1E).

All eight animals had similar peak levels of SIV RNA in plasma and CSF before the initiation of ART as well as similar numbers of circulating CD4 T cells and monocytes (Table 1). At necropsy (terminal time point), the untreated animals displayed a clinical phenotype similar to that of untreated HIV-1 patients (47). Three of four animals had fewer than 400 CD4 T cells/μl of blood and increased numbers of monocytes compared to the ART-suppressed group (Table 1; see Fig. S1 in the supplemental material). Additionally, the CD4 T cells in all four ART-treated animals rebounded to preinfection levels after ART initiation (Fig. S1).

10.1128/mBio.01659-19.1 FIG S1 CD4, CD8, and monocyte percent change over time in ART-treated and untreated SIVmac251-infected macaques. Whole-blood FACS was conducted on blood from all animals in the study throughout the course of infection and ART treatment. The red lines display SIV-infected untreated animal data, and the blue lines display SIV-infected ART-suppressed animal data. The arrow indicates the start of ART. Download FIG S1, PDF file, 0.1 MB. Copyright © 2019 Abreu et al. 2019 Abreu et al. This content is distributed under the terms of the Creative Commons Attribution 4.0 International license.

Mathematical modeling of the decay of plasma and CSF SIV RNA was done by fitting data to a four-parameter, two-exponential model as previously described (3, 48, 49). In plasma, this analysis revealed a biphasic decay consistent with observations made in HIV-1-infected individuals (Fig. 1C) (49). Viral decay in all animals had half-lives that were also similar to those previously reported for HIV-1-infected individuals (49), with the first-phase half-lives ranging from 0.5 to 0.7 days and the second-phase half-lives ranging from 13.75 to 19.4 days. In contrast, the four SIV-infected untreated animals experienced only a slight decay in viremia from peak at day 14 p.i. to a set point viremia that was maintained above 10^6^ copies/ml (Fig. 1A).

The mathematical modeling of CSF decay was similar to that in plasma in two of the four ART-treated animals (Rh402 and Rh403) that displayed biphasic decay with first-phase half-lives of 0.83 and 0.6 days, respectively (Fig. 1F). The second-phase half-lives were 18.6 and 14.7 days, respectively. The additional suppressed animals (Rh404 and Rh405) had lower peak viremia in CSF, resulting in fewer time points with measurable virus above the ddPCR limit of quantitation. Therefore, these animals have reported half-lives only for the first phase of decay, 1.2 and 0.3 days, respectively. Together, these data suggest that the ART-suppressed SIVmac251-infected rhesus macaque model mirrors HIV-1-infected individuals after ART initiation in both plasma and CSF and provides an appropriate model for the study of HIV-1 latency.

### Quantitation of SIV RNA and DNA in tissues of untreated and ART-suppressed macaques.

**FIG 2:**
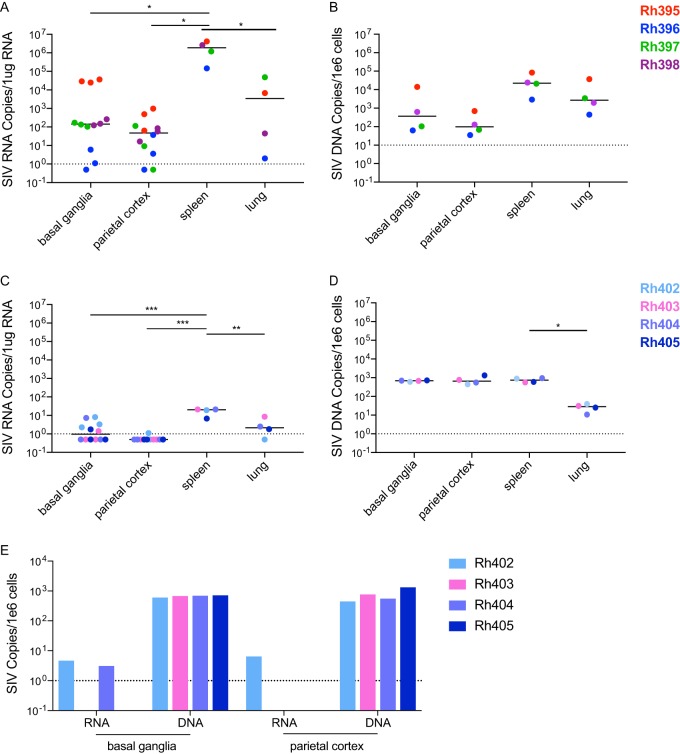
Detection of SIV RNA and DNA in tissues of untreated and ART-suppressed SIVmac251-infected macaques. SIV RNA and DNA were measured in independent tissue samples from the brain s(basal ganglia and parietal cortex), spleens, and lungs from untreated animals (A and B) and ART-treated animals (C and D). Select samples from the brain were used to measure SIV RNA and DNA simultaneously in ART-treated animals (E) to determine if provirus was present despite the absence of SIV RNA. Multiple measurements from the same animal and same tissue were averaged. Statistics were then calculated using a one-way ANOVA with Tukey’s multiple-comparison test. Values that are significantly different are indicated by a bar and asterisks as follows: *, *P* < 0.05; **, *P* < 0.01; ***, *P* < 0.001.

SIV RNA and DNA were quantitated for the first time in the SIVmac251 model in the spleen, lung, and brain for both untreated and ART-suppressed macaques. Because of the multifocal nature of HIV and SIV replication in the brain, SIV RNA was measured in triplicate samples from two separate regions (basal ganglia and parietal cortex). SIV RNA and DNA were detected in all tissues in all untreated macaques. The brain had significantly lower levels of SIV RNA compared to the spleen (*P* < 0.0001) and similar levels compared to the lung (Fig. 2A; see also Table S1 in the supplemental material). SIV DNA was measured in one 4-mm sample punch per tissue per animal. There was no significant difference in DNA levels in all tissues tested (Fig. 2B; Table S1).

10.1128/mBio.01659-19.6 TABLE S1 Levels of SIV RNA and DNA in macaque tissues at necropsy (terminal time point). Download Table S1, PDF file, 0.01 MB. Copyright © 2019 Abreu et al. 2019 Abreu et al. This content is distributed under the terms of the Creative Commons Attribution 4.0 International license.

SIV RNA was quantified in at least one brain sample from all four ART-suppressed macaques, although at very low levels (median of 2 copies per μg RNA; range, 1 to 4.5 copies per μg RNA; Fig. 2C and Table S1). SIV RNA was detected predominantly in the basal ganglia and in the parietal cortex of only one animal, in contrast to previous studies using dual-inoculated pigtailed macaques in which SIV was detected in both regions during ART suppression (3, 48, 50). For one replicate, RNA and DNA were isolated from the same sample of basal ganglia and parietal cortex so that SIV RNA and DNA levels could be directly compared on a per cell basis. All ART-suppressed macaques had low copy numbers of SIV RNA but higher levels of SIV DNA. These data demonstrate that ART reduces SIV RNA expression to very low levels (less than 5 copies per million cells [Fig. 2E]) despite significant levels of SIV DNA (median, 700 copies per million cells [Fig. 2E]) in both basal ganglia and parietal cortex.

SIV RNA was also measured in the spleens and lungs of the ART-suppressed animals (Fig. 2C; Table S1) with median values of 20 and 2.5 copies per 1 μg RNA, respectively. The expression level of SIV RNA in these tissues was significantly higher than the SIV RNA levels in the brain (*P* < 0.0001, Fig. 2C, Table S1). Despite the difference between SIV RNA in the tissues and brain, there were equivalent levels of SIV DNA detected in basal ganglia, parietal cortex, and spleen (Fig. 2D; Table S1). These findings agree with our previously published work which found that early treatment of SIV infection leads to high levels of SIV DNA in the brain despite control of RNA replication (51). These data demonstrate that SIV DNA persists in all tissues examined and that SIV RNA is also detected in tissues at low levels during ART suppression even when virus is not detected in plasma.

### Quantification of cellular SIV DNA and RNA levels in CD11b^+^ macrophages and CD4^+^ T cells isolated from tissues of ART-suppressed SIVmac251-infected macaques.

**FIG 3:**
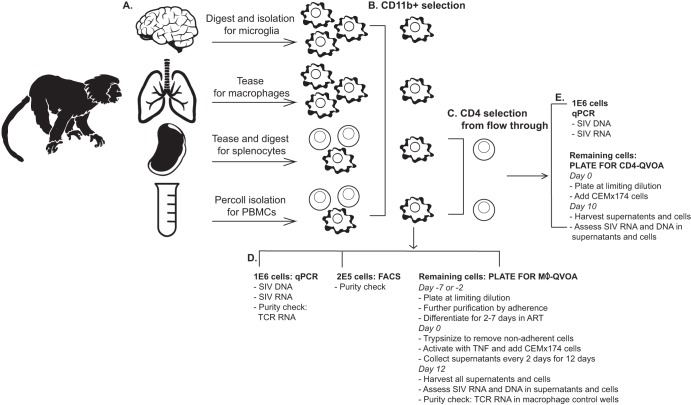
Schematic of cell isolation methods and purity assessments for the macrophage and CD4 QVOAs. (A) Single cells were isolated from brain, lung, spleen, and blood from four SIV-infected ART-suppressed macaques. (B) Monocytes from blood and tissue macrophages from brain, lung, and spleen were purified from single-cell suspensions using CD11b-positive selection. (C) Flowthrough samples from the CD11b selection were then used to isolate untouched CD4 T cells. (D) CD11b^+^ cells were saved for purity checks by qPCR and FACS assessment as well as SIV RNA and DNA measurements. For further purification by adherence, the remaining CD11b^+^ cells were plated at limiting dilutions in the presence of ART and allowed to differentiate for 2 to 7 days, depending on tissue of origin. Nonadherent cells and ART were removed prior to activation with TNF and coculture with CEMx174 cells. Supernatants were sampled every 2 days for 12 days. On day 12, all supernatants and cells were harvested and assessed for the presence of SIV RNA and DNA, as well as T cell contamination by TCRβ RNA. (E) CD4 T cells were saved for SIV RNA and DNA measurements by qPCR. The remaining CD4 cells were plated at limiting dilutions and cocultured with CEMx174 cells for 10 days. On day 10, all supernatants and cells were harvested and assessed for the presence of SIV RNA and DNA.

**FIG 4:**
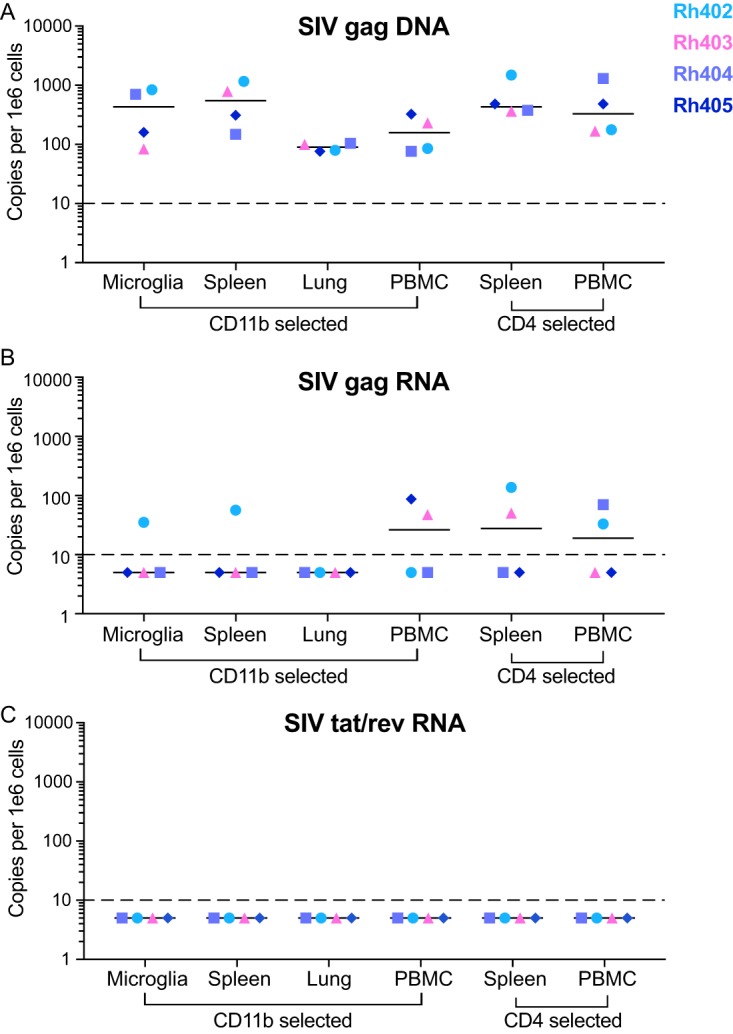
Comparison of cellular SIV DNA and RNA levels in CD11b^+^ macrophages and CD4^+^ T cells isolated from tissues of ART-suppressed SIVmac251-infected macaques. CD11b^+^ cells were isolated from brains, spleens, lungs, and PBMCs, and CD4^+^ T cells were isolated from spleens and PBMCs from four SIV-infected ART-suppressed macaques. Cellular DNA and RNA were then extracted and analyzed for SIV gag DNA (A), SIV gag RNA (B), and SIV tat/rev RNA (C) by qPCR. The dashed line represents the limit of quantification (LOQ) for each qPCR assay.

It is remarkable that the amounts of SIV DNA measured in spleens and brains of ART-suppressed animals were very similar (Fig. 2D; Table S1), since the spleen contains a large number of CD4 T cells and macrophages, which are both targets of HIV and SIV infection. In contrast, microglia and perivascular macrophages are the major target of HIV and SIV infection in the brain. Therefore, to determine the contribution of specific cell types to the levels of SIV DNA expression in tissues and blood, we isolated CD4 T cells from peripheral blood mononuclear cells (PBMCs) and spleen and CD11b^+^ cells from PBMCs, spleen, brain, and lung. In an effort for clarity, we have designed a schematic that illustrates how these samples were obtained and how the isolated cells were used throughout the study (Fig. 3). CD11b^+^ is a known marker for myeloid cells and has been used by our group for previous studies because it reliably selects myeloid cells from multiple tissues in the macaque (3–5) (Fig. S3 and S4 ). Using quantitative PCR (qPCR), we measured the level of SIV DNA in isolated CD4 T cells and CD11b^+^ cells (Fig. 4A; Table S2). SIV DNA was quantifiable in CD4 T cells and CD11b^+^ cells isolated from all tissues. The levels of SIV DNA quantified in both CD4 T cells and CD11b^+^ cells isolated from the same tissue were equivalent (Fig. 4A, Spleen and PBMC). Additionally, the amount of SIV DNA measured per million cells was equivalent to the amount of SIV DNA measured in whole tissue.

10.1128/mBio.01659-19.3 FIG S3 Representative FACS plot of CD11b staining in whole blood from rhesus macaques. Singlets are removed, and samples are gated to remove debris based on forward scatter (FSC) versus side scatter (SSC) (not shown). Whole blood is gated based on CD11b expression (A). CD11b-positive cells are gated on TLR2 expression (B). TLR2^+^ cells are gated for CD14 and CD16 expression (C). CD11b^+^ TLR2^−^ cells are gated for CD159a and CD3 expression (D), and CD159a^−^ CD3^−^ cells are then gated for CD20 expression (E). CD11b^−^ cells are gated for TLR2 expression (F) and are negative. TLR2^−^ CD11b^−^ cells are gated for CD159a and CD3 expression (G), and CD159a^−^ CD3^−^ cells are then gated for CD20 expression (H). Download FIG S3, PDF file, 0.6 MB. Copyright © 2019 Abreu et al. 2019 Abreu et al. This content is distributed under the terms of the Creative Commons Attribution 4.0 International license.

10.1128/mBio.01659-19.4 FIG S4 Representative gating strategy and purity assessment for CD11b selected cells from PBMCs and spleen. Representative gating strategy and purity assessment for CD11b selected cells from PBMCs (A to E) and spleen (F to J). Singlets are removed (not shown), and samples are gated to remove debris based on FSC versus SSC. (A and F). Samples are then gated to remove cells positive for Live/Dead stain (B and G). Live cells are then gated to assess CD11b and CD3 based on no-stain controls (C and H). CD11b and CD3 percentages are gated before selection (D and I) and after CD11b selection (E and J). Download FIG S4, PDF file, 0.6 MB. Copyright © 2019 Abreu et al. 2019 Abreu et al. This content is distributed under the terms of the Creative Commons Attribution 4.0 International license.

10.1128/mBio.01659-19.7 TABLE S2 SIV gag DNA, gag, and tat/rev RNA measurements in isolated cells. Download Table S2, PDF file, 0.02 MB. Copyright © 2019 Abreu et al. 2019 Abreu et al. This content is distributed under the terms of the Creative Commons Attribution 4.0 International license.

In addition to measuring SIV DNA, SIV gag RNA and SIV tat/rev RNA were quantitated to confirm that the isolated cells were latent and that detection of virus in the subsequent QVOA would be from reactivation of latent genomes. In contrast to quantitation of DNA, the majority of samples were negative for SIV gag RNA and all samples were negative for tat/rev RNA (Fig. 4B and C; Table S2). The samples that were positive for SIV gag RNA had very low levels of RNA detected (range, 30 to 130 copies per million cells). Detection of SIV gag RNA does not necessarily indicate active transcription as does the presence of the SIV tat/rev RNA (52, 53). These results suggest that the residual SIV gag RNA detected in the isolated CD11b^+^ cells and CD4 T cells in suppressed macaques is not indicative of active replication but reflects the expected stochastic oscillations in HIV and SIV expression during suppressive ART (54). Therefore, detection of virus in the QVOA would measure reactivation of latent viral genomes.

### Quantitation of functional latent reservoirs in CD4 T cells in blood and spleen.

Quantitation of the CD4 T cell latent reservoir has rarely been done in SIV macaque models, including SIVmac251, despite the availability of a SIV-specific CD4 T cell quantitative viral outgrowth assay (QVOA) (1, 39, 55, 56). Therefore, to characterize the latent reservoir in this model, CD4 T cells were isolated from PBMCs and spleen and assessed by the previously published SIV-specific CD4 T cell QVOA (Fig. 3) (4, 39, 55, 56).

**FIG 5:**
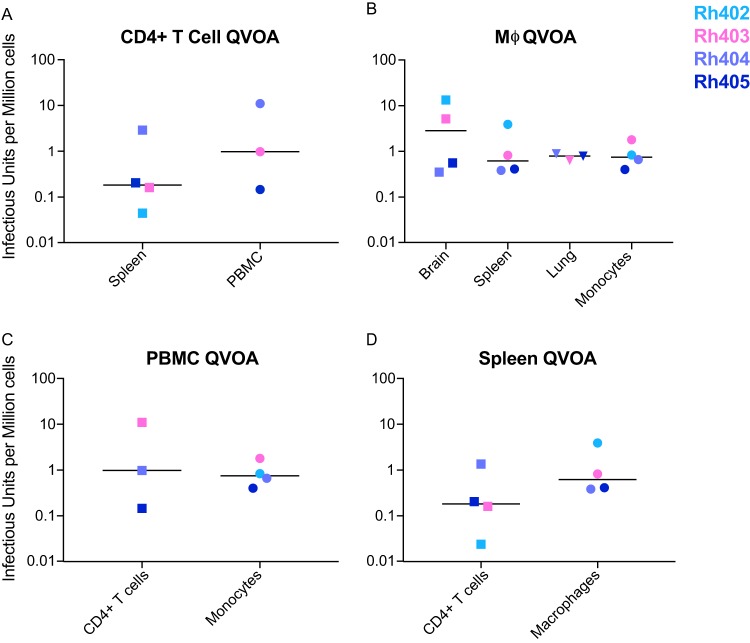
Functional latent reservoirs detected in CD4 T cells, monocytes, and macrophages isolated from SIVmac251-infected ART-suppressed macaques. Infectious units per million cells (IUPMs) were calculated for CD4 T cell (A) and monocyte/macrophage (B) QVOAs. Cells were isolated from blood, spleen, lung, and brain from ART-treated SIV-infected macaques and plated at limiting dilutions. Supernatants were sampled every 2 days and measured for SIV RNA. Comparison of blood CD4 T cell and monocyte-derived macrophage (MDM) (C) and splenic CD4 T cell and macrophage (D) IUPM values. Samples with IUPM values below the limit of detection are not shown. (A and C) Rh402 CD4 from PBMCs, (B) Rh402 macrophages from lung and B cell QVOAs from all animals.

Three of the four ART-suppressed SIVmac251-infected macaques had detectable frequencies of CD4 T cells harboring replication-competent virus in blood (Fig. 5A and Table 2). Animals Rh403, Rh404, and Rh405 had between 0.15 to 11 infectious units per million (IUPM) with a median value of 1 IUPM, which is similar to the frequency of functional latently infected CD4 T cells in blood from HIV-1-infected ART-suppressed individuals (2). The animal (Rh402) with an undetectable IUPM had limited sample available for CD4 isolation. The well with the most cells contained only 0.5 million cells, which is near the limit of detection in the current assay (Fig. 5A and Table 2). Results from the CD4 QVOA in the ART-suppressed SIVmac251-infected macaque model, therefore, parallel the level of functional latency reported for HIV-1-infected ART-suppressed individuals as measured by similar QVOA assays. The spleens from all four ART-suppressed animals had CD4 T cells (CD4s) harboring replication-competent virus, varying from an IUPM of 0.04 to 2.9, with a median value of 0.2 (Fig. 5A and Table 2). The frequencies of latently infected CD4s in spleen and blood were not significantly different (*P* = 0.53), a finding that is consistent with previously reported observations in HIV-1-infected individuals when comparing blood and lymph node CD4 reservoir levels (57). The total number of cells assessed for each CD4 QVOA and the limit of detection for each individual assay are shown in Table S3.

**TABLE 2:** IUPM values calculated for SIVmac251-infected ART-suppressed macaques

Animal	IUPM^*a*^							
	CD4^+^ T cell QVOA		Macrophage QVOA				B cell QVOA	
	Spleen	PBMCs	Brain	Spleen	Lung	PBMCs	Spleen	PBMCs
Rh402	0.04	<LOD	13.4	3.92	<LOD	0.83	<LOD	<LOD
Rh403	0.16	11.01	5.14	0.82	0.64	1.80	<LOD	<LOD
Rh404	2.89	0.98	0.35	0.38	0.89	0.66	<LOD	<LOD
Rh405	0.15	0.15	0.56	0.41	0.79	0.40	<LOD	<LOD

a IUPM, infectious units per million cells.

10.1128/mBio.01659-19.8 TABLE S3 Total number of cells assessed and IUPM limits of detection for all QVOA assays. Download Table S3, PDF file, 0.02 MB. Copyright © 2019 Abreu et al. 2019 Abreu et al. This content is distributed under the terms of the Creative Commons Attribution 4.0 International license.

### Blood monocytes from ART-suppressed macaques harbor latent SIV genomes.

Monocytes are constantly egressing from the bone marrow and stay in circulation for an average of 3 days before they egress into tissues to become long-lived tissue macrophages (58, 59). Our previous studies using the MΦ-QVOA in the dual-inoculated SIV-infected pigtailed macaque model have demonstrated that monocytes harbor replication-competent SIV and produce virus when allowed to mature to macrophages in culture (4). This finding corroborates studies of other lentiviruses, such as visna virus, in which blood monocytes are also latently infected and do not produce virus until the cells are allowed to mature to macrophages (60, 61). Therefore, throughout this study, the term monocyte will refer to monocyte-derived macrophages (MDMs) in culture.

To determine whether latently infected monocytes were present in ART-suppressed SIVmac251-infected macaques, CD11b^+^ blood cells were isolated and then, after plating at limiting dilutions, allowed to differentiate for 7 days in the presence of combined ART; monocyte-derived macrophages were then activated with TPP (tumor necrosis factor alpha [TNF-α], Pam3CSK4, and prostaglandin) and cocultured in the presence of CEMx174 cells, as previously described (Fig. 3) (4). Cell supernatants were harvested every 2 days and assessed for SIV RNA by qRT-PCR. Cell isolation purities were assessed prior to plating by fluorescence-activated cell sorting (FACS) (Table S4), and a representative FACS plot can be found in Fig. S4. A T cell receptor beta (TCRβ) qRT-PCR assay was used to assess the possible contribution of CD4 T cell-derived virus to the MΦ-QVOA (Table S5) (4). Additionally, the number of cells assessed per MΦ-QVOA and the limit of detection for each assay are given in Table S3.

10.1128/mBio.01659-19.9 TABLE S4 Cell purities after selection before Mϕ and B cell QVOA plating. Download Table S4, PDF file, 0.01 MB. Copyright © 2019 Abreu et al. 2019 Abreu et al. This content is distributed under the terms of the Creative Commons Attribution 4.0 International license.

10.1128/mBio.01659-19.10 TABLE S5 Calculated probabilities of infected CD4^+^ T cell contribution to Mϕ-QVOA results. Download Table S5, PDF file, 0.02 MB. Copyright © 2019 Abreu et al. 2019 Abreu et al. This content is distributed under the terms of the Creative Commons Attribution 4.0 International license.

Monocytes isolated from PBMCs had IUPMs ranging from 0.4 to 1.8 with a median of 0.75 IUPM (Fig. 5B), which was very similar to the median IUPM of 1 in CD4 T cells, isolated from the PBMCs of the same animals (*P* = 0.42; Fig. 5C and Table 2). Based on the levels of TCRβ RNA in the samples, the numbers of CD4 T cells in the cultures were negligible and could not account for the SIV RNA observed (0.000001% chance of an infected CD4 per well; Table S5). Additionally, when comparing the number of cells assessed and limits of detection for the CD4 T cell QVOA and MΦ-QVOA, there is no statistical difference between the two assays (Table S3 and data not shown). Thus, similar to the recently described dual-inoculated SIV model, monocytes harbor replication-competent virus in ART-suppressed SIVmac251-infected macaques (5).

### Quantitation of functional latent reservoirs in tissue macrophages.

In tissue, long-lived macrophages are made up of both monocytes that traffic into tissues and differentiate into monocyte-derived macrophages and resident tissue macrophages that are derived from embryonic progenitors (58, 59, 62). The embryonal macrophages are long-lived and self-replenish, while the half-life of MDMs varies from a few days up to several months, and the MDMs are reseeded by the blood (59). We will refer to both of these populations as tissue macrophages. We have previously shown in the dual-inoculated SIV-infected pigtailed macaque model that tissues such as spleen and lung contain large numbers of SIV-infected macrophages (3). Therefore, we hypothesized that these organs may also harbor latently infected macrophages in the SIVmac251 model. Tissue macrophages were isolated from spleen and lung using magnetic bead isolation for the marker CD11b as previously described (3), and MΦ-QVOA was conducted as described above. The purity of cell isolations was assessed prior to plating by FACS (Table S4 and Fig. S4), and the number of cells assessed per MΦ-QVOA and the limit of detection for each assay are shown in Table S3. Similarly, a TCRβ qRT-PCR was performed to assess the potential contribution of CD4 T cells to the virus detected in the macrophage cultures (Table S5) (3, 4).

All ART-suppressed animals had macrophages harboring replication-competent virus in their spleens, and three of the four ART-suppressed animals had them in lungs (Fig. 5B and Table 2). Macrophages isolated from spleen had IUPMs ranging from 0.4 to 3.9 with a median value of 0.6, which was similar to the median IUPM of 0.2 in CD4 T cells isolated from the spleens of the same animals (*P* = 0.43; Fig. 5D). These results suggest that both CD4 T cells and myeloid cells have the potential of contributing equally to the SIV reservoir in blood and spleen, resulting in approximately 1 in 1 million cells in blood and 1 in 5 million cells in spleen being latently infected. Three of four ART-suppressed animals had detectable frequencies of latently infected macrophages in lung (Fig. 5C and Table 2). Animals Rh403, Rh404, and Rh405 had IUPMs ranging from 0.6 to 0.9 with a median value of 0.8, suggesting that 1 in 1.25 million lung macrophages harbors replication-competent virus. The animal (Rh402) with an undetectable IUPM had fewer macrophages available after isolation to perform the assay. The well with the most cells contained only 0.9 million cells, near the limit of detection of the assay. Based on the levels of TCRβ RNA in samples from both spleen and lung, the number of CD4 T cells in the cultures was negligible and could not account for the SIV RNA levels observed (spleen 0.00002% chance and lung 0.000001% chance of an infected CD4 per well [Table S5]). Thus, the large number of macrophages that become infected during acute infection have the potential to become latently infected cells upon ART intervention, contributing to the overall size of the viral reservoir.

### Quantitation of functional latent reservoirs in the CNS.

We have previously demonstrated that brain macrophages are a functional latent reservoir in ART-suppressed SIV-infected pigtailed macaques, a SIV model with consistent CNS infection and development of encephalitis (3, 4). However, CNS infection has not been previously characterized in the SIVmac251 macaque model without immune manipulation. Therefore, we examined whether brain macrophages in the ART-suppressed SIVmac251-infected macaque model also harbor a functional latent reservoir.

All ART-suppressed SIVmac251-infected macaques had detectable frequencies of brain macrophages harboring replication-competent virus. Brain macrophages, consisting of both perivascular macrophages (derived from blood monocytes) and resident microglia, had IUPMs ranging from 0.35 to 13.4 with a median value of 2.9, suggesting that 1 in 300,000 cells harbor latent replication-competent virus (Fig. 5B). The MΦ-QVOA values from brain macrophages had the greatest variability in IUPM values compared to other tissue macrophages and monocytes. However, the highest IUPM values correlated with higher CSF viral loads during acute infection prior to ART initiation as well as higher levels of SIV RNA in brain tissue at the terminal time point (Fig. S5). Our previous studies reported a strong correlation between the viral loads in CSF and levels of SIV RNA in brains from SIV-infected untreated macaques (63) and demonstrated that increased levels of the proinflammatory proteins CCL2 and interleukin 6 (IL-6) in the CSF predict CNS disease (64, 65). Therefore, these factors may also influence the size of the latent reservoir in the brain. Additionally, based on the levels of TCRβ RNA in samples from brain, the number of CD4 T cells in the cultures was negligible and could not account for the SIV RNA levels observed (0.00007% chance of an infected CD4 per well [Table S5]).

10.1128/mBio.01659-19.5 FIG S5 Correlations between IUPM values measured in brain and SIV RNA in CSF and brain. IUPM values measured from brain macrophages were correlated with peak viral load values measured in the CSF (A) and terminal time point levels of SIV RNA measure in brain (B). *R* squared values were calculated using the linear regression analysis provided by Prism 7. Download FIG S5, PDF file, 0.1 MB. Copyright © 2019 Abreu et al. 2019 Abreu et al. This content is distributed under the terms of the Creative Commons Attribution 4.0 International license.

### B cell control to assess contribution of CD4 T cells to the MΦ-QVOA.

To assess whether CD3 contamination was a source of reactivated virus measured in our MΦ-QVOAs, we assessed B cells (not a target of HIV or SIV infection) isolated from PBMCs and spleens in the QVOA assay. CD20^+^ cells were isolated from the PBMCs of three out of four ART-suppressed macaques and from the spleens of all four ART-suppressed macaques using the same positive selection method used for the CD11b^+^ cells. The purity after selection was confirmed by FACS (Table S4), and the levels of CD3 cells were similar to those measured in the CD11b^+^ selection method (Table S4). After selection, the cells were plated at limiting dilutions following the CD4 QVOA assay protocol (see above). We chose to follow the conditions that were most favorable for CD4 T cells because this would allow any contaminating cells that harbored replication-competent virus to reactivate and replicate successfully. The number of B cells assessed and limit of detection for each assay are listed in Table S3. All B cell QVOAs were negative (Table 2). These data, in conjunction with other controls, strongly support that the level of CD3 cells measured in the MΦ-QVOA after selection do not contribute to the positive values obtained in our assay.

### Viruses produced in MΦ-QVOAs were capable of establishing *de novo* infection.

**FIG 6:**
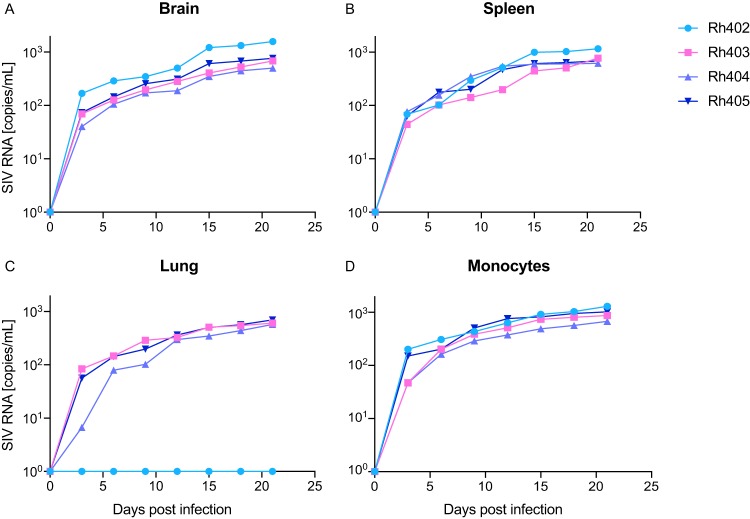
Macrophage-produced virus is capable of establishing *de novo* infection. Activated CD4 T cells from a healthy rhesus macaque were spinoculated with culture supernatant from brain macrophages (A), splenic macrophages (B), lung macrophages (C), and monocyte-derived macrophages (D) QVOAs. All infections were normalized, One hundred copies of gag RNA as measured by RT-qPCR was used for the initial input. After spinoculation, the cells were washed and cultured for 21 days. Supernatant samples were taken every 3 days and measured for SIV RNA.

To confirm that the viruses measured in MΦ-QVOAs were replication competent and capable of producing *de novo* infection, we infected activated CD4 T cells isolated from a healthy rhesus macaque with supernatant collected from MΦ-QVOA wells and assessed viral kinetics. Viruses from all tissue MΦ-QVOAs were able to replicate exponentially in activated CD4 T cells (Fig. 6). Virus produced by macrophages isolated from spleen, lung, brain, and blood from three ART-suppressed animals (Rh403, Rh404 and Rh405) were all capable of infecting and expanding in activated CD4 T cells. One animal (Rh402) had detectable IUPMs only in spleen, brain, and blood MΦ-QVOAs, and therefore, only the supernatants from these MΦ-QVOAs were infectious. The supernatant from the negative lung MΦ-QVOA was used as a negative control (Fig. 6C). These data suggest that macrophages contain a long-lived reservoir that is capable of reestablishing infection upon reactivation.

This study has characterized both the CD4 T cell and macrophage reservoirs in ART-suppressed SIVmac251-infected rhesus macaques. The level of functional latency in CD4 T cells in this SIV model closely mirrors the level of latently infected CD4 T cells in ART-suppressed HIV-1-infected individuals. In addition, the quantitation of a functionally latent reservoir in monocytes and tissue macrophages provides an SIV model to study functional latency and eradication in both cell types, including those cells bearing latent but replication-competent SIV in the CNS.

## DISCUSSION

SIVmac251 infection of rhesus macaques is a well-characterized animal model for AIDS research, used in more than 500 published studies on HIV-1 pathogenesis, vaccine development, immune responses during infection, molecular studies of virus replication, and strategies for viral control. With the advances in HIV-1 cure research, full characterization of viral reservoirs during ART is a pivotal step in the development of efficacious strategies to achieve a functional cure, making the SIVmac251 rhesus macaque model a valuable tool to investigate viral latency in tissues and cells that are not readily accessible in HIV-1-infected individuals.

Therefore, it is important to systematically quantify the latent viral reservoir in the SIVmac251 model during ART, since it is widely used for HIV studies to eradicate or fully suppress latency as well as for HIV vaccine development. Using QVOAs specific for CD4 T cells and myeloid cells developed in our laboratory, our results show that the CD4 T cell functional latent reservoirs in PBMCs and spleens of ART-suppressed SIVmac251-infected macaques were present at the same frequency (approximately one latently infected CD4 cell per million) as those in ART-suppressed HIV-1-infected individuals and SIV-infected pigtailed macaques (1, 2, 5, 39). Surprisingly, we found that within the same animal, the frequencies of latently infected blood monocytes and spleen macrophages were similar to those of latently infected CD4 T cells in the blood and spleen, respectively. This requires further investigation, as these similar levels of latency in cell types of a different lineage may shed light on the mechanism of HIV latency as a whole. Additionally, replication-competent SIVmac251 was found in the brains of all ART-suppressed macaques, and the frequency of latently infected brain macrophages (microglia and perivascular macrophages) was comparable to the frequency of infected spleen and lung macrophages. These results are very similar to those we recently published, which assessed the viral reservoir in peripheral tissues and brains of ART-suppressed SIV-infected pigtailed macaques, a dual-inoculation model in which untreated animals progress rapidly to AIDS and have a high frequency of SIV encephalitis (3, 5).

The detection of SIVmac251 functional latent genomes in monocytes and tissue macrophages is novel. Monocytes play an important role in immune surveillance and can enter tissues and mature into macrophages. The short half-life on monocytes suggests that these cells cannot represent a viral reservoir. However, entry of latently infected monocytes into tissues that mature into long-lived macrophages would constitute a reservoir. Interestingly, monocytes are known to harbor both HIV-1 and SIV genomic DNA (66–68), and monocytes were the only blood cells carrying HIV-1 genomes in the “Mississippi baby” (69). Monocytes may become infected during egression from the bone marrow, which would suggest a novel reservoir in that compartment and a biomarker role for monocytes. Another possible explanation is that monocytic progenitor cells in the bone marrow may be latently infected, similarly to what has been observed in other lentiviruses such as visna virus (70). This hypothesis is supported by the surface expression of CD4 on promonocytes (71) and on promonocytic cell lines, which have been reported to be susceptible to strains of HIV (72). In addition, myeloid progenitor cells are known to be susceptible to other viruses that induce latency, such as human herpesvirus 6 (HHV-6) (73), which may be kept quiescent by the regulatory T cell-driven immune suppressive microenvironment in bone marrow (74, 75).

The quantification of latently infected tissue macrophages, identified as functional latent reservoirs in this study, has direct impact in HIV cure strategies. There are conflicting studies in the HIV and SIV fields that either point to macrophages as a bona fide reservoir or completely discount the cell type. It was recently reported that resident urethral macrophages isolated from penile tissue contained latent HIV and constitute a novel reservoir for the virus (76). However, previous work suggested that though macrophages harbor HIV DNA in the liver, the viral DNA was unable to productively infect activated CD4 T cells (77). These studies point to the possibility that a functional macrophage reservoir may not be present in every tissue but also highlight the difficulty of working with primary tissues isolated from humans. Additionally, though it is widely accepted that cell-to-cell contact is a more efficient mechanism of infection, whether this is the only mode of macrophage infection remains controversial. Some studies suggest that while phagocytosis of infected CD4 T cells is more efficient, other methods of infection can occur (78, 79). Furthermore, the same study that showed that macrophages can become infected via phagocytosis of CD4 T cells in an *in vitro* setting also demonstrated that the presence of ART prevents this infection from occurring and leads to degradation of viral DNA within 72 h (78). These data suggest that our method, where all macrophages isolated from tissue or blood are kept in ART for a minimum of 72 h prior to activation, would prevent spread of infection by phagocytosis of infected CD4 T cells. Finally, SIV macaque models have been previously used to study latent SIV reservoirs as a model for HIV-1. The majority of these studies have measured SIV DNA and RNA but have not measured functionally latent cells by QVOA or similar assays. In addition, other animal models, such as BLT mice reconstituted with either lymphocytes or monocytes and suppressed on ART, have been used to study HIV-1 latency and also have demonstrated that both cell types harbor latent HIV-1 (80, 81).

This study may not answer the controversy concerning HIV macrophage latency; however, it does provide compelling evidence that SIV latency extends well beyond the CD4 T cell reservoir with myeloid cells representing another substantial reservoir in the SIVmac251 model. The different biological functions of CD4 T cells and myeloid cells as well as sites of latency, may require different eradication strategies in order to target the two cell types. HIV latency in ART-suppressed HIV-infected individuals needs to be rigorously examined in monocytes and tissue macrophages to ascertain whether there is another functional latent reservoir in HIV.

## MATERIALS AND METHODS

### Development of SIVmac251 stock.

A sample of the original SIVmac251 viral stock produced by the laboratory of Ronald Desrosiers was expanded by infecting rhesus macaque peripheral blood mononuclear cells (PBMCs) (82). Half-volume medium changes were performed two or three times per week, and the supernatants were tested for the capsid protein p27. Supernatant from peak infection was aliquoted to produce a stock that is similar in p27 levels and infectious titer (50% tissue culture infective dose [TCID_50_]) to the original Desrosiers’ stock (82). Additionally, our expanded SIVmac251 strain was sent to Shelby O’Connor of the Department of Pathology and Laboratory Medicine at the University of Wisconsin—Madison for deep sequencing, and the viral genome was confirmed by BLAST to be similar to previously published SIVmac251strains (see Fig. S2 in the supplemental material) (83, 84).

10.1128/mBio.01659-19.2 FIG S2 Tree of SIVmac251 full-length sequences. The tree was constructed using the maximum likelihood algorithm via Geneious. Each symbol represents one SIVmac251 sequence submitted to the NCBI database from four labs as follows: Barouch lab sequences (red triangles), Desrosiers lab sequences (orange diamonds), Miller lab sequences (blue squares), JHU Retrovirus lab sequence (black circle). Download FIG S2, PDF file, 0.02 MB. Copyright © 2019 Abreu et al. 2019 Abreu et al. This content is distributed under the terms of the Creative Commons Attribution 4.0 International license.

### Animal studies.

Eight juvenile male rhesus macaques (Macaca mulatta) who were negative for Mamu-A*01, Mamu-B*08, and Mamu-B*13 were inoculated intravenously with SIVmac251with 20 AID_50_ (50% animal infectious dose) (calculated following a previously described method [82]). Beginning at day 14 postinoculation, four of eight macaques (Rh402, Rh403, Rh404, and Rh405) were treated once daily with a subcutaneous injection of 2.5 mg of dolutegravir (ViiV) per kg of body weight, 20 mg/kg PMPA, and 40 mg/kg FTC (Gilead). Cerebrospinal fluid (CSF) and blood samples were collected three times before inoculation to obtain baseline values. Samples were then collected at days 7, 10, 14, 21, and 28 and every 2 weeks thereafter until 3 months, followed by once monthly collection until euthanasia at approximately 130 days postinoculation.

### Whole-blood cell counts.

Whole-blood samples were stained with pretitrated amounts of monoclonal antibodies using 100 μl of whole blood at room temperature for 20 min. The antibody panels consisted of anti-CD3 V500 (clone SP34-2; BD Biosciences), anti-CD4 BV650 (clone OKT4; BioLegend), anti-CD8a BV570 (clone RPA-T8; BioLegend), anti-TLR2 AF488 (clone T2.5; BioLegend), anti-CD14 BV 650 (clone M5E2; BD Biosciences), and anti-CD16 AF700 (clone 3G8; BioLegend). Whole-blood samples were then lysed and fixed in 2 ml of FACS Lysing Solution (BD Biosciences, San Jose, CA) for 10 min at room temperature. Samples were collected in a centrifuge at 400 × *g* for 5 min, washed in 2 ml of 1× phosphate-buffered saline (PBS), and then resuspended in 0.5 ml of PBS for analysis. Flow cytometry was performed on a BD LSRFortessa (BD Biosciences, San Jose, CA). Data were analyzed using FlowJo 10.0.8 software (FlowJo, LLC, Ashland, OR).

### Quantitation of SIV gag RNA.

Viral RNA was measured in the plasma samples, CSF samples, cell culture supernatants, and tissues by quantitative reverse transcription-PCR (qRT-PCR) or digital droplet PCR (ddPCR) as previously described (56, 66, 83, 85). In brief, viral RNA was isolated in duplicate from 140 μl of plasma or supernatant using the QIAamp Viral RNA Minikit (Qiagen, Valencia, CA, USA) according to the manufacturer’s protocol. For tissues, total RNA was isolated from 50 mg of tissue in singlet or triplicate using the RNeasy kit (Qiagen) according to the manufacturer’s protocol. As suggested by the manufacturer’s protocol, an on-column DNase digestion was performed for all samples using the RNase-free DNase kit (Qiagen) and 3 U of RQ1 DNase (Promega, Madison WI), and the columns were incubated at room temperature for 20 min. Quantification of SIV *gag* RNA was performed by RT-qPCR using the QuantiTect Virus kit (Qiagen) or ddPCR using the One-Step RT ddPCR Adv kit for probes (Bio-Rad) and a primer/probe set for SIV *gag*: SIV21F, 5′-GTCTGCGTCATCTGGTGCATTC-3′; SIV22R, 5′-CACTAGGTGTCTCTGCACTATCTGTTTTG-3′; SIV23, FAM-5′-CTTCCTCAGTGTGTTTCACTTTCTCTTCTG-3′-BH1 (Integrated DNA Technologies, Coralville, IA, USA). A Roto-Gene Q thermocycler (Qiagen) was used for qRT-PCRs, and the Bio-Rad QX-100 system was used for ddPCR reactions, as previously described (83).

### Quantification of cellular SIV tat/rev RNA.

Quantification of SIV tat/rev RNA was performed as previously described (5). In brief, all samples were assessed by RT-qPCR using the QuantiTect Virus kit (Qiagen) and primer/probe set. The primer/probe set used were as follows: SIV tat/rev forward primer, 5′-CGMARGAGAAGAAGAACTCCGAARAAG-3′; SIV tat/rev reverse primer, 5′-CTATCTGYCAAGGCCARGA-3′, probe, FAM5′-AACCAGAGAAGGMRAAGAAGGAGACGGTGM-3′ BH1 (Integrated DNA Technologies, Coralville, IA, USA). Three reactions were performed for each sample. To control for DNA contamination, one reaction was analyzed without reverse transcriptase. Reactions were analyzed using the CFX96 Real-Time PCR Detection System (Bio-Rad) as follows: 30 min at 50°C, 5 min at 95°C, and 40 cycles, with 1 cycle consisting of 15 s at 95°C, 30 s at 54°C, and 1 min at 60°C.

### Quantitation of SIV DNA.

DNA was isolated from tissues using the All prep kit (Qiagen) according to the manufacturer’s recommendations. Viral DNA was measured in tissues by multiplex qPCR with the MP kit (Qiagen) or ddPCR with the Supermix for Probes kit (Bio-Rad) using primers in the SIV gag region and macaque beta interferon (IFN-β) for sample normalization and cellular quantitation. A Roto-Gene Q thermocycler (Qiagen) was used for qPCR reactions, and the Bio-Rad QX-100 system was used for ddPCR reactions, as previously described (3).

### Mathematical modeling of decay in viremia with ART.

The decay of viremia in plasma and viral load in CSF following the initiation of ART were evaluated using a two-exponential model as described previously (3, 48, 49). For each animal, the measurements at each time point were used to generate a four-parameter fit to the two-exponential equation of the form: *V*(*t*) *= V*_0_ [*Ae^–μ1t^ + Be^–μ2t^*], where *V*_0_ is the pretreatment viral load. This results in a biphasic decay if *μ_1_ ≠ μ_2_*; a biphasic decay is a better fit for the data here, as shown previously in ART-treated HIV patients (49), where an initial brief but fast decline in viral load is followed by a slower decline. In addition to identifying the decay parameters for each animal, for each animal cohort, we generated the geometric mean of the data for each time point and performed the same fit for this cohort mean. The parameter fits were obtained using the trust region reflective algorithm (*lsqnonlin*) in MATLAB (MathWorks, Natick MA). For experimental data points that were below the limit of detection, we applied a no-cost function penalty if the predicted viral level at that time point was also under the limit of detection; if the predicted viral level was above the limit, the square of the error between the predicted level and the limit of detection was added to the cost function. This enabled us to include the below-limit points in the parameterization without artificially allocating them an arbitrary value. We report the half-lives of the two decay phases as follows: *t*_1/2_ *=* ln(2)*/*μ*_i_*. *A* and *B* represent proportionality constants for the contribution of the two phases to the overall decline of viral levels; differences in these parameters are typically less insightful than differences in the decay rates μ.

### Isolation of cells from lung and spleen.

Cells were isolated from lung and spleen as previously described (4). In brief, lung cells were mechanically separated from freshly excised tissues using an 18-gauge needle and scalpel. Fresh spleen was minced in cold PBS using scalpels, followed by digestion using collagenase and DNase to remove macrophages. Both lung and spleen samples were passed through a 100-μm-mesh cell strainer in cold RPMI to obtain single-cell suspensions. As needed, tissue samples were lysed using red blood lysis buffer. CD4 T cells and myeloid cells were isolated as described in the QVOA method sections below.

### Isolation of brain macrophages.

Brain macrophages were isolated from excess sections of frontal, parietal, and temporal cortices and from basal ganglia and thalamus as previously described (3). In brief, perfused tissue was stripped of meninges and vesicles, washed with phosphate-buffered saline, and then digested for 30 min in trypsin-DNase digestion solution (Dulbecco’s modified Eagle medium [DMEM] supplemented with 0.25% trypsin, 50 μg DNase/ml, and 50 mg gentamicin/ml) at 37°C with agitation. Digested tissue was filtered through 183-μm sterile mesh, followed by 100-μm sterile filter, washed once with DMEM containing 10% fetal bovine serum (DMEM-10% FBS) and then pelleted. Cells were resuspended in PBS, mixed with Percoll, and centrifuged at 411,000 × *g* for 30 min at room temperature. Brain macrophages were harvested from the gradient layer below the myelin cap and pelleted in DMEM-10% FBS. Cells were counted and further purified as described in the QVOA assay below.

### CD4 T cell QVOA.

The levels of latently infected CD4 T cells in blood and spleen were assessed by CD4 T cells QVOA as previously described (4, 39). In brief, bulk CD4 T cells were isolated using the negative selection kit (Miltenyi) and plated in fivefold limiting dilutions. Cells were activated by being cocultured with CEMsx174 cells for 10 days. On day 10, supernatants were harvested and assessed for RNA, and the frequency of cells harboring replication-competent virus were determined as previously described (86).

### Monocyte/macrophage quantitative viral outgrowth assay (MΦ-QVOA).

MΦ-QVOAs were conducted on monocytes and macrophages isolated from PBMCs, brain, spleen, and lung as previously described (3). It should be noted that all MΦ-QVOAs were conducted on myeloid cells isolated from fresh tissue. In our experience, myeloid cells do not survive the freeze-thaw process well. In brief, monocytes and macrophages were purified using a nonhuman primate CD11b antibody-conjugated microbead kits (Miltenyi Biotec, Auburn, CA) according to the manufacturer’s recommendation and assessed for purity by FACS analysis (CD3 SP34-2 Biolegend; CD11b Bear1 Beckman Coulter). The wells on the plates were coated with poly-l-lysine solution (Sigma) for at least 30 min and washed twice with PBS prior to cell plating. Purified monocytes and macrophages were cultured in duplicate in 10-fold limiting dilutions in the presence of 10 μM zidovudine (Sigma) 5 nM raltegravir (Merck), and 25 nM darunavir (Janssen, Titusville, NJ). Macrophages isolated from brain, spleen, and lung were incubated for 2 to 4 days to allow for adherence. Monocytes isolated from PBMCs were incubated for 7 days to allow for macrophage differentiation. The cells were then washed twice with PBS to remove nonadherent cells and replenished with activation medium containing 10 ng/ml tumor necrosis factor (TNF) (ProSpec), 1 μg/ml Pam3CSK4 (Sigma), and 1** **μg/ml prostaglandin (Sigma), called TPP. Between 10^5^ and 10^4^ CEMx174 cells were added to all wells except TCRβ controls, as previously described (3, 4). Supernatants were collected and replenished with TNF, Pam3CSK4, and prostaglandin E2 every 2 days and assessed for SIV RNA by qRT-PCR.

Supernatant from early time points (days 4, 6, and 8) and supernatant from later time points (days 10, 12, and 14) were each pooled and assessed for viral RNA as described above. Cells were collected at day 14 and lysed for RNA and DNA as described above. The frequency of cells harboring replication-competent virus was determined using the IUPMStats v1.0 infection frequency calculator and expressed as infectious units per million (IUPM) (86)). All MΦ-QVOAs were assessed for CD3^+^ T cell contamination using qRT-PCR for *TCRβ* as previously described (3, 4).

### B cell control QVOA.

QVOAs were conducted on B cells isolated from PBMCs and spleens from SIV-infected ART-suppressed macaques. B cells were isolated using an anti-CD20 biotinylated antibody (clone 2H7; Biolegend), antibiotin antibody (EasySep), and EasySep magnetic nanoparticles. The CD20 selection was performed following the same method as the CD11b isolations previously described (3–5). In brief, cells were thawed, counted, brought up in 50 μl of selection buffer (2% FBS, 1 mM EDTA in 1× PBS), and incubated with anti-CD20 antibody for 20 min at 4°C. The cells were then washed and brought up in 100 μl/10^7^ cells of selection buffer and incubated with the EasySep antibiotin antibody for 15 min at 4°C. EasySep magnetic particles were then added and incubated for 10 min at 4°C. Magnetically labeled cells were then removed using the EasySep EasyEights magnet. The cells were washed, counted, and saved for FACS purity analysis (CD3 SP34-2 [BioLegend]; CD20 2H7 [BioLegend]) and plated at limiting dilutions following the CD4 QVOA protocol as described above and previously (39).

### *In vitro* infection of PBMCs.

PBMCs from uninfected rhesus macaques were isolated by Percoll density gradient and cultured in R10-IL-2 medium (RPMI supplemented with 10% FBS, 2 mM glutamine, 100 μg·ml^−1^ penicillin-streptomycin, 2 μg/ml recombinant human interleukin 2 [IL-2] [Life Technologies, Inc.]) and activated with 2 μg/ml phytohemagglutinin (PHA) (Life Technologies, Inc.) for 72 h. CD4 T cells were isolated from PHA-activated PBMCs using the negative CD4 isolation kit (Miltenyi) and subjected to spinoculation for 2 h with 500-μl supernatant containing 100 copies of SIV gag RNA from positive MΦ-QVOA wells. Supernatants were collected at days 0, 3, 6, 9, 12, 15, 18, and 21 after spinoculation. RNA was isolated from supernatant, and SIV RNA was quantitated by qRT-PCR.

### Statistics.

All statistical analyses were done using a one-way analysis of variance (ANOVA) with Tukey’s multiple-comparison test.

### Study approval.

All animal work was approved by the Johns Hopkins University Institutional Animal Care and Use Committee and determined to be in accordance with the guidelines outlined in the Animal Welfare Act and Regulation (87) and the *Guide for the Care and Use of Laboratory Animals* (88).
