# Supplementary figures and images for: Myeloid and CD4 T Cells Comprise the Latent Reservoir in Antiretroviral Therapy-Suppressed SIVmac251-Infected Macaques
Source: mBio. 2019 Aug 20;10(4):e01659-19. doi: 10.1128/mBio.01659-19 (PMC6703426; doi:10.1128/mBio.01659-19)

**CD4+ T cells**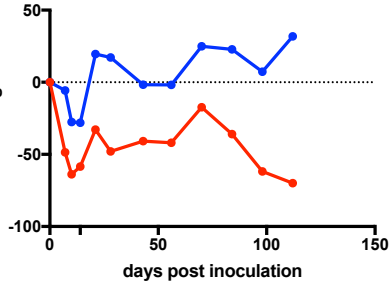**CD8+ T cells**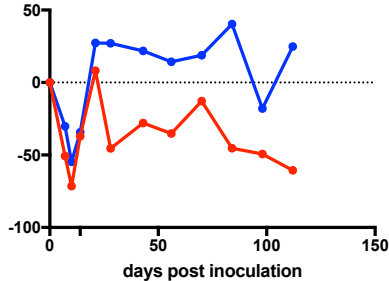**monocytes**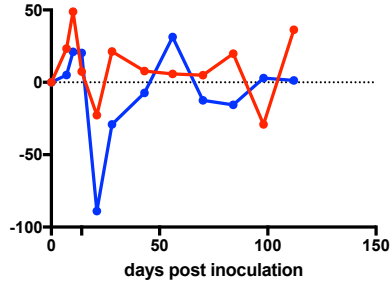

—●— Rh251  
—●— Rh251 + ART

Supplement: FIG S1 [file mBio.01659-19-sf001.pdf]

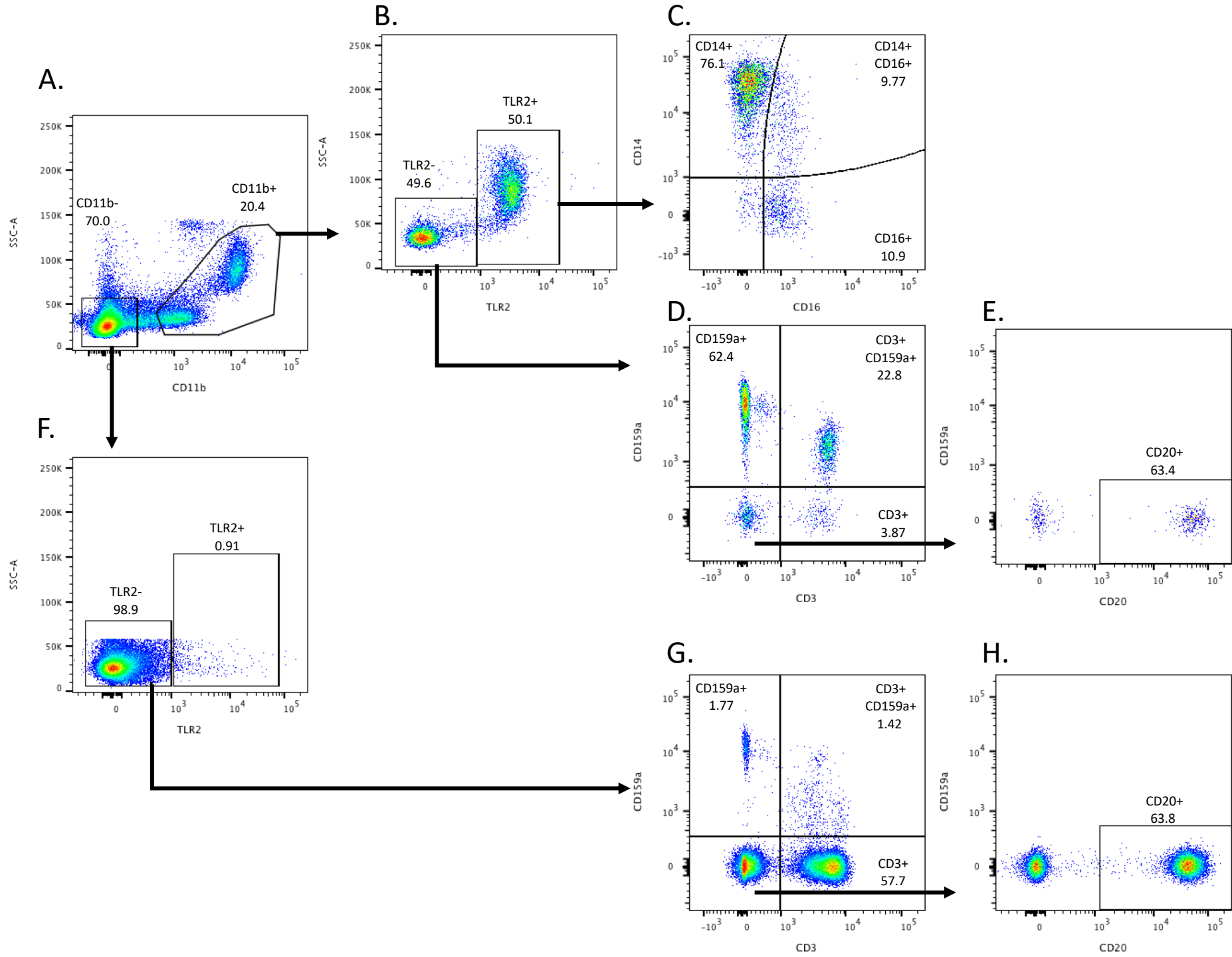

Supplement: FIG S3 [file mBio.01659-19-sf003.pdf]

PBMC

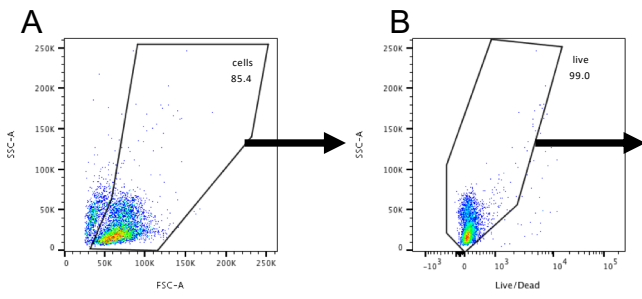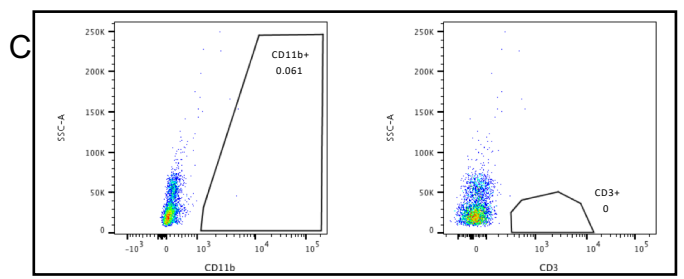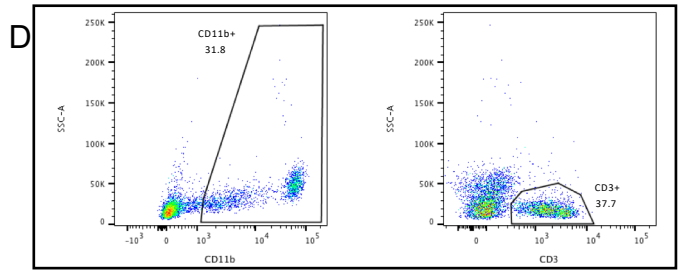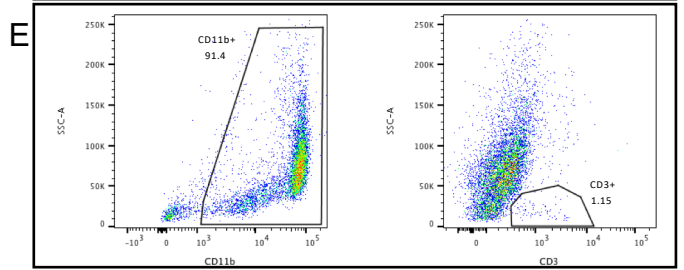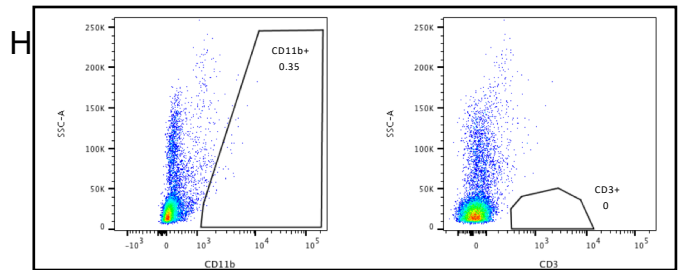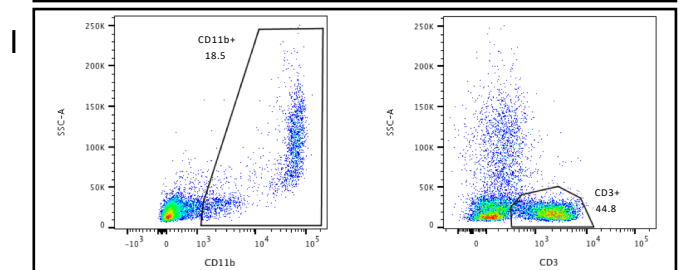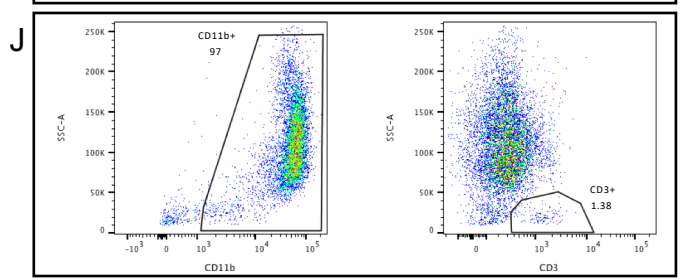

Spleen

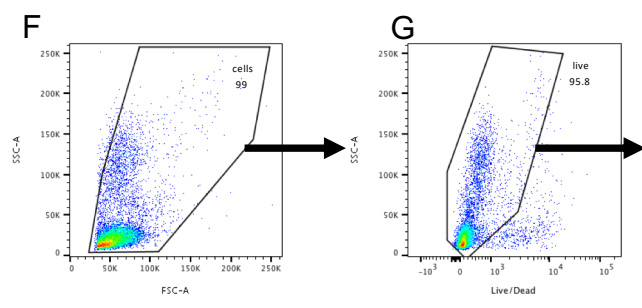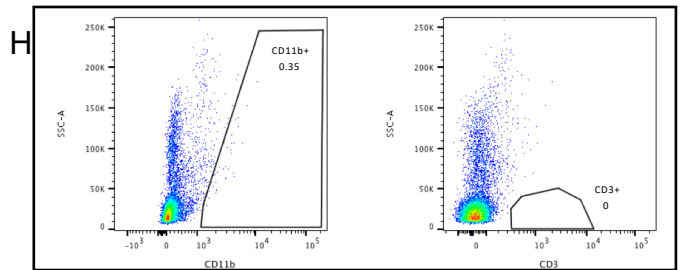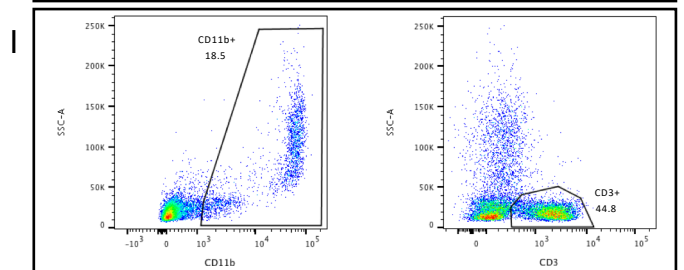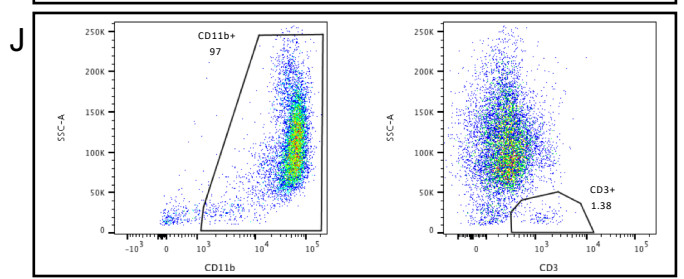

Supplement: FIG S4 [file mBio.01659-19-sf004.pdf]

A

**Brain IUPM and CSF peak VL**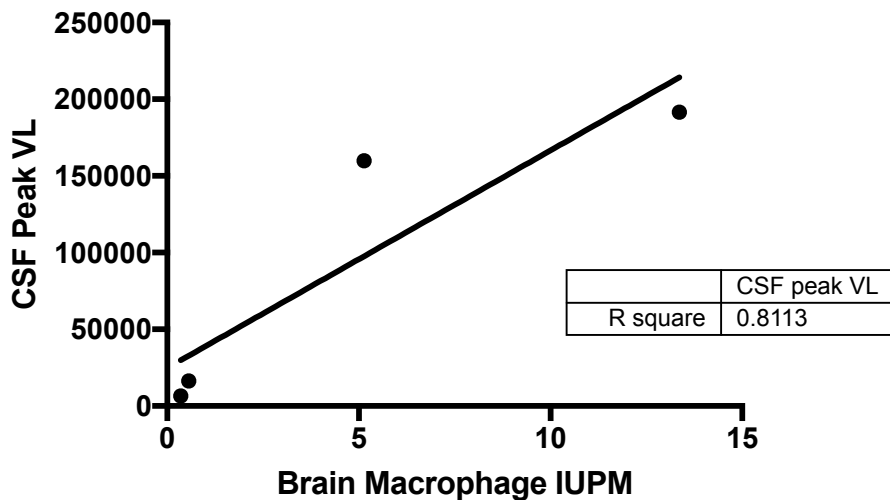

B

**Brain IUPM and Brain SIV RNA**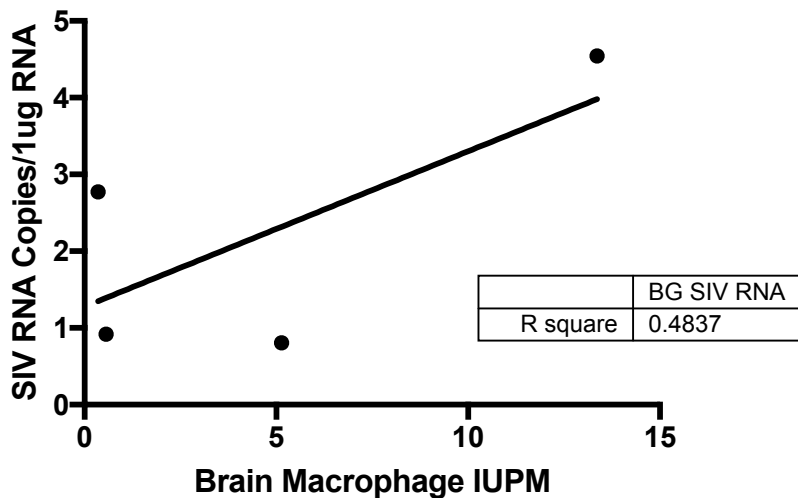

Supplement: FIG S5 [file mBio.01659-19-sf005.pdf]

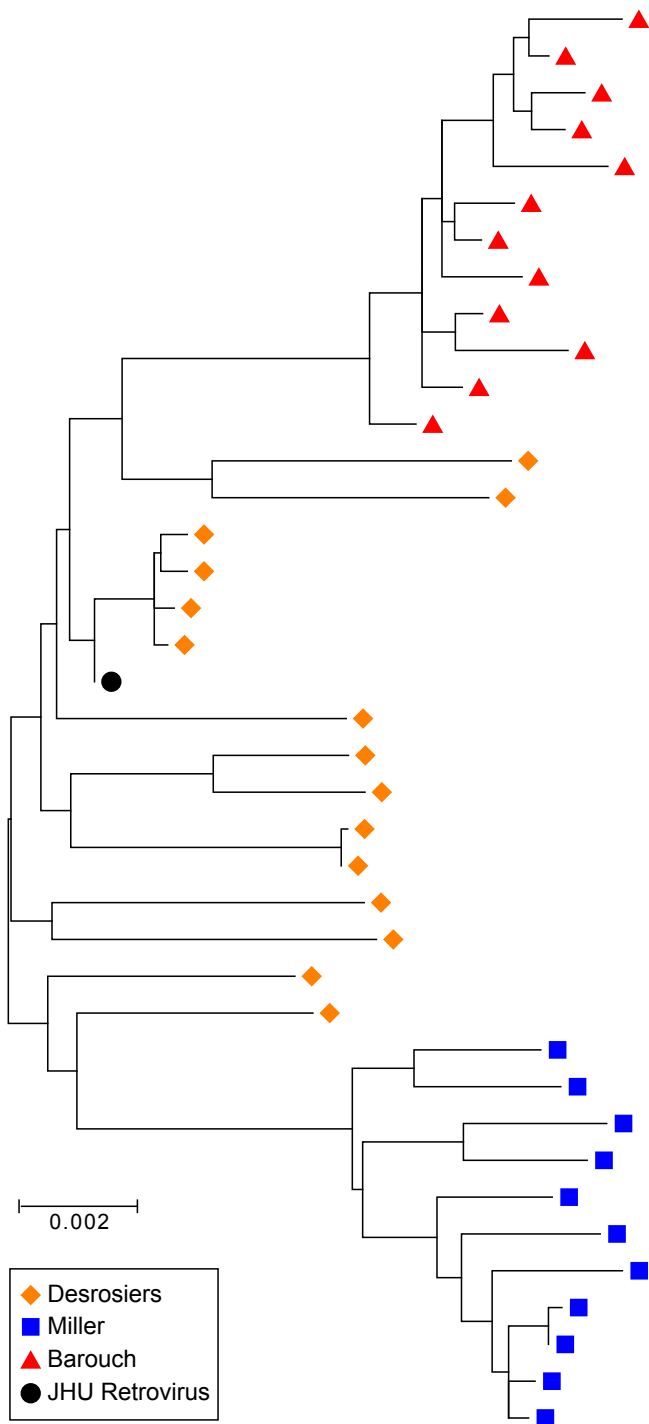

Supplement: FIG S2 [file mBio.01659-19-sf002.pdf]
