# Supplementary material for: Myeloid and CD4 T Cells Comprise the Latent Reservoir in Antiretroviral Therapy-Suppressed SIVmac251-Infected Macaques
Source: mBio. 2019 Aug 20;10(4):e01659-19. doi: 10.1128/mBio.01659-19 (PMC6703426; doi:10.1128/mBio.01659-19)
Supplement: TABLE S1 [file mBio.01659-19-st001.pdf]

Supplemental Table 1. Levels of SIV RNA and DNA in macaque tissues at necropsy (terminal timepoint)

Animal identifier	Group	RNA levels in Tissue (SIV copies/ug)				DNA levels in Tissue (SIV copies/1e6 cells)			
		Basal Ganglia	Parietal Cortex	Lung	Spleen	Basal Ganglia	Parietal Cortex	Lung	Spleen
**Rh395**	untreated	3.0E+04	511	6.8E+03	4.1E+06	1.4E+04	693	8.3E+04	3.7E+04
**Rh396**	untreated	2	13	2	1.5E+06	63	35	2.9E+03	449
**Rh397**	untreated	136	41	4.8E+04	1.2E+06	104	67	2.1E+04	3.4E+03
**Rh398**	untreated	178	53	44	2.6E+06	624	129	2.4E+04	1.9E+03
**Rh402**	ART suppressed	4.5	1	<LOD	19	604	447	39	887
**Rh403**	ART suppressed	1	<LOD	8	21	679	765	31	561
**Rh404**	ART suppressed	3	<LOD	2	21	698	553	11	942
**Rh405**	ART suppressed	1	<LOD	2	7	721	1325	25	602
