# Supplementary material for: Myeloid and CD4 T Cells Comprise the Latent Reservoir in Antiretroviral Therapy-Suppressed SIVmac251-Infected Macaques
Source: mBio. 2019 Aug 20;10(4):e01659-19. doi: 10.1128/mBio.01659-19 (PMC6703426; doi:10.1128/mBio.01659-19)
Supplement: TABLE S2 [file mBio.01659-19-st002.pdf]

Supplemental Table 2. SIV gag DNA, gag and tat/rev RNA measurements in isolated cells

Assay	Cells Isolated	Animal ID Rh402	Rh403	Rh404	Rh405
SIV gag DNA (copies/1e6 cells)	CD11b Microglia	839	83	704	160
	CD11b Spleen	1158	788	149	310
	CD11b Lung	79	99	104	76
	CD11b PBMC	85	230	76	325
	CD4 Spleen	1482	362	376	483
	CD4 PBMC	177	167	1301	483
SIV gag RNA (copies/1e6 cells)	CD11b Microglia	35	<LOD	<LOD	<LOD
	CD11b Spleen	57	<LOD	<LOD	<LOD
	CD11b Lung	<LOD	<LOD	<LOD	<LOD
	CD11b PBMC	<LOD	48	<LOD	87
	CD4 Spleen	137	50	<LOD	<LOD
	CD4 PBMC	33	<LOD	70	<LOD
SIV Tat/Rev RNA (copies/1e6 cells)	CD11b Microglia	<LOD	<LOD	<LOD	<LOD
	CD11b Spleen	<LOD	<LOD	<LOD	<LOD
	CD11b Lung	<LOD	<LOD	<LOD	<LOD
	CD11b PBMC	<LOD	<LOD	<LOD	<LOD
	CD4 Spleen	<LOD	<LOD	<LOD	<LOD
	CD4 PBMC	<LOD	<LOD	<LOD	<LOD

LOD - Limit of detection equals 10 copies per reaction
