# Supplementary material for: Myeloid and CD4 T Cells Comprise the Latent Reservoir in Antiretroviral Therapy-Suppressed SIVmac251-Infected Macaques
Source: mBio. 2019 Aug 20;10(4):e01659-19. doi: 10.1128/mBio.01659-19 (PMC6703426; doi:10.1128/mBio.01659-19)
Supplement: TABLE S3 [file mBio.01659-19-st003.pdf]

Supplemental Table 3. Total number of cells assessed and IUPM limits of detection for all QVOA assays

QVOA and Animal ID	PBMC		Spleen		Lung		Microglia	
	Cell # in assay	LOD	Cell # in assay	LOD	Cell # in assay	LOD	Cell # in assay	LOD
**CD4 T cell QVOA**								
Rh402	1.25E+07*	0.06*	2.87E+07	0.02	NA	NA	NA	NA
Rh403	3.75E+06	0.18	2.50E+07	0.03	NA	NA	NA	NA
Rh404	8.25E+06	0.08	3.75E+07	0.02	NA	NA	NA	NA
Rh405	8.75E+06	0.08	6.25E+06	0.11	NA	NA	NA	NA
**Mϕ QVOA**								
Rh402	6.13E+06	0.11	1.31E+07	0.05	2.15E+06*	0.32*	3.84E+06	0.18
Rh403	2.84E+06	0.24	6.22E+06	0.11	8.04E+06	0.09	9.99E+05	0.69
Rh404	7.73E+06	0.09	1.33E+07	0.05	5.78E+06	0.12	1.44E+07	0.05
Rh405	1.27E+08	0.01	1.24E+07	0.06	6.44E+06	0.11	9.20E+06	0.08
**B cell QVOA**								
Rh402	2.1E+06*	0.33*	2.4E+06*	0.29*	NA	NA	NA	NA
Rh403	1.0E+06*	0.69*	2.0E+06*	0.34*	NA	NA	NA	NA
Rh404	2.4E+06*	0.29*	2.0E+06*	0.34*	NA	NA	NA	NA
Rh405	NA	NA	2.5E+06*	0.28*	NA	NA	NA	NA

* Negative QVOA result

NA – sample not available

LOD – limit of detection

IUPM – infectious units per million
