# Supplementary material for: Myeloid and CD4 T Cells Comprise the Latent Reservoir in Antiretroviral Therapy-Suppressed SIVmac251-Infected Macaques
Source: mBio. 2019 Aug 20;10(4):e01659-19. doi: 10.1128/mBio.01659-19 (PMC6703426; doi:10.1128/mBio.01659-19)
Supplement: TABLE S4 [file mBio.01659-19-st004.pdf]

Supplemental Table 4. Cell purities post-selection pre Mϕ and B cell QVOA plating

Cell type and Animal ID	PBMC		Spleen		Lung	
**CD11b purities post selection**						
	% CD3+	% CD11b+	% CD3+	% CD11b+	% CD3+	% CD11b+
Rh402	2.2	87.9	5.2	89.8	1.3	92.8
Rh403	19.1	69.7	9.1	84.1	5.6	91.4
Rh404	1.2	91.4	1.4	97.0	1.6	96.2
Rh405	0.9	87.1	4.9	93.0	0.4	97.0
**CD20 purities post selection**						
	% CD3+	% CD20+	% CD3+	% CD20+	% CD3+	% CD20+
Rh402	0.3	94.8	2.7	88.4	NA	NA
Rh403	6.9	87.9	1.5	96.9	NA	NA
Rh404	0.6	96.9	3.6	92.7	NA	NA
Rh405	NA	NA	4.5	92.2	NA	NA

NA – sample not available
