# Supplementary material for: Myeloid and CD4 T Cells Comprise the Latent Reservoir in Antiretroviral Therapy-Suppressed SIVmac251-Infected Macaques
Source: mBio. 2019 Aug 20;10(4):e01659-19. doi: 10.1128/mBio.01659-19 (PMC6703426; doi:10.1128/mBio.01659-19)
Supplement: TABLE S5 [file mBio.01659-19-st005.pdf]

Supplemental Table 5. Calculated probabilities of infected CD4+ T cell contribution to Mϕ-QVOA results

Compartment and animal identifier	IUPM for CD4+ T cells	# CD3+ T cells in Mϕ-QVOA (via TCRβ RNA)	% CD3+CD4+ cells in blood at necropsy (via FACs)	# CD4+ T cells in Mϕ-QVOA	probability of an infected CD4+ T cell present in Mϕ-QVOA	percent chance an infected CD4+ T cell is present in Mϕ-QVOA	IUPM for CD11b+ macrophages
**Lung***							
**Rh402**	<LOD	0.0	60.1	0.0	0.00E+00	0.00E+00	<LOD
**Rh403**	1	0.0	57.7	0.0	0.00E+00	0.00E+00	0.64
**Rh404**	11	0.0	44.1	0.0	0.00E+00	0.00E+00	0.88
**Rh405**	0.153999	0.0	79.5	0.0	0.00E+00	0.00E+00	0.79
**PBMC**							
**Rh402**	<LOD	0.0	60.1	0.0	0.00E+00	0.00E+00	0.83
**Rh403**	1	0.0	57.7	0.0	0.00E+00	0.00E+00	1.8
**Rh404**	11	0.0	44.1	0.0	0.00E+00	0.00E+00	0.66
**Rh405**	0.153999	0.0	79.5	0.0	0.00E+00	0.00E+00	0.4
**Spleen**							
**Rh402**	0.03	0.0	60.1	0.0	0.00E+00	0.00E+00	3.92
**Rh403**	0.27	2.2	57.7	1.3	9.67E-07	9.67E-05	0.82
**Rh404**	0.18	1.0	44.1	0.4	1.63E-07	1.63E-05	0.38
**Rh405**	0.22	0.0	79.5	0.0	0.00E+00	0.00E+00	0.41
**Brain***							
**Rh402**	<LOD	0.0	60.1	0.0	0.00E+00	0.00E+00	13.36
**Rh403**	1	4.9	57.7	2.8	2.82E-06	2.82E-04	5.14
**Rh404**	11	0.0	44.1	0.0	0.00E+00	0.00E+00	0.35
**Rh405**	0.153999	0.0	79.5	0.0	0.00E+00	0.00E+00	0.56

* Indicates blood CD4 IUPMs used in place of tissues because CD4s were not isolated
